# Hepatitis C Treatment: current and future perspectives

**DOI:** 10.1186/1743-422X-7-296

**Published:** 2010-11-01

**Authors:** Saira Munir, Sana Saleem, Muhammad Idrees, Aaliyah Tariq, Sadia Butt, Bisma Rauff, Abrar Hussain, Sadaf Badar, Mahrukh Naudhani, Zareen Fatima, Muhmmad Ali, Liaqat Ali, Madiha Akram, Mahwish Aftab, Bushra Khubaib, Zunaira Awan

**Affiliations:** 1 Division of Molecular Virology & Molecular Diagnostics, National Centre of Excellence in Molecular Biology, University of the Punjab, Lahore, Pakistan

## Abstract

Hepatitis C virus (HCV) is a member of *Flaviviridae *family and one of the major causes of liver disease. There are about 175 million HCV infected patients worldwide that constitute 3% of world's population. The main route of HCV transmission is parental however 90% intravenous drug users are at highest risk. Standard interferon and ribavirin remained a gold standard of chronic HCV treatment having 38-43% sustained virological response rates. Currently the standard therapy for HCV is pegylated interferon (PEG-INF) with ribavirin. This therapy achieves 50% sustained virological response (SVR) for genotype 1 and 80% for genotype 2 & 3. As pegylated interferon is expensive, standard interferon is still the main therapy for HCV treatment in under developed countries. On the other hand, studies showed that pegylated IFN and RBV therapy has severe side effects like hematological complications. Herbal medicines (laccase, proanthocyandin, Rhodiola kirilowii) are also being in use as a natural and alternative way for treatment of HCV but there is not a single significant report documented yet. Best SVR indicators are genotype 3 and 2, < 0.2 million IU/mL pretreatment viral load, rapid virological response (RVR) rate and age <40 years. New therapeutic approaches are under study like interferon related systems, modified forms of ribavirin, internal ribosome entry site (HCV IRES) inhibitors, NS3 and NS5a inhibitors, novel immunomodulators and specifically targeted anti-viral therapy for hepatitis C compounds. More remedial therapies include caspase inhibitors, anti-fibrotic agents, antibody treatment and vaccines.

## Background

Hepatitis C virus (HCV) is a meticulous factor of liver disease and one of the most important health issues worldwide [1,2]. Hepatitis C has approximately 175 million Global Disease Burden which represent almost 3% of the whole population in the world, each year 3 to 4 million new patients with HCV are diagnosed. HCV remains endemic in many countries of the world [3-5]. Statistics based on general healthy population revealed that HCV has 5.3% seroprevalence in Pakistan, 2.2% in Turkey and 7.7% in Zimbabwe [6-8]. Hepatitis C virus infection is not a main factor of mortality in the first decade of infection [9]. Even though, the biological aspects of HCV are revealed to a great extent in recent years, an absolute therapy of hepatitis C remains problematic in a large majority of patients [10] and about 50% HCV patients does not attain sustained virological Responses [11-13].

A few years back, it was not easy to study HCV in invitro because there was no proficient system present but fortunately Heller *et al *got success in establishing in vitro model of HCV virions. This system proves good for high level production and secretion of HCV virions hence this system expands the scope of tools present for HCV study [14,15]. Many patients remain asymptomatic for years and are only detected on health screening or at the time of blood transfer [16]. Peg. INF and ribavirin therapy is still the therapy of choice for HCV patients besides having many side affects [17,12]. As HCV is mainly a chronic disease and progress very slowly therefore persistent infection is a typical characteristic of disease which can be found in approximately 75% patient at primarily stage. Prospective studies conducted on natural history suggest that HCV take almost 20 years to develop cirrhosis and only 20% of cirrhotic patient can develop Hepatocellular Carcinoma (HCC) after 40 years of preliminary infection [18,10].

### HCV genotypes and treatment response

Patients with different HCV genotypes react in a different way to alpha interferon because genotype is one of the strongest prognostic aspects of sustained virological response [19,20]. This clinical importance of HCV genotype was revealed by clinical studies based on interferon treatment response account [5]. Patients show more sustained virological response when suffered from HCV genotype 2 and 3 as compared to HCV infected persons of genotype1 [6]. Patients infected with HCV genotype 2 and 3 show 65% SVR and patients with HCV genotype 1 show 30% Sustained Virological Response (SVR) [7,8]. Thus genotype of patients must not be over looked when giving standard interferon therapy. Different ethnic groups respond differently to standard therapy of HCV and hence there is variation in Early Treatment Response (ETR) and SVR rates [21].

### Mechanism of Pathogenesis and interferon resistance

Now a number of mechanisms associated with escape of the pathogen from the host's immune response, hepatocyte damage and molecular oncogenesis of hepatocellular carcinoma have been elucidated. Inefficient clearance of virus from patient's body is basically due to the hyper-variability of virus envelope protein that enables HCV to neutralize antibody [22,23]. Once the virus enters the hepatocytes through receptor mediated endocytosis and starts replication, it initiate damaging of hepatocyte, the major component of which is through the host's own immune response [24,23]. Interferon is the most potent natural weapon of the host against intra-cellular viral infection. HCV, however, owing to intricate actions of its genomic proteins is equipped with ability to evade the natural interferon-mediated clearance. HCV core protein has been reported to decrease the robustness of the host's immune response by decreasing transcription of interferon induced antiviral genes [25,23]. HCV NS3/4A protease also has been concerned in inhibiting the interferon amplification loop which otherwise results in suppression of HCV replication. Inhibition of HCV protease can reverse the effects of HCV infection that make protease inhibitors one of the most noteworthy potential therapeutic agents for HCV [26,25].

### Route of transmission and treatment response

At first, it was believed that most frequent route of transmission of HCV was blood transfusion and intravenous drug abuse. But recent epidemiological studies suggest further routes of transmission [27]. The main route of HCV transmission is parental. However 90% intravenous drug users are at highest risk of getting HCV infection such as those who require multiple blood transfusions and blood products (hemophiliacs) or those who go through major surgery [28,29]. Unlike HBV, HCV infection transfer less frequently by sexual or intimate contact (0.4 to 3%). Domestic contacts are also at low risk [30]. Almost 5% HCV infections are caused by needle stick injury [29,30]. 3% to 5% infants acquire HCV from infected mother by perinatal transmission [31]. HCV is present in saliva and milk but transfer of HCV infection through breast milk has not been reported [32,33].

Community barbershops also play a key role in HCV transmission in under development countries [27]. Some other reported risk factors of disease transmission are dental and surgical treatments, circumcision, ear piercing, tattooing and dialysis [34-36]. In a study conducted on 3351 patients of HCV in Pakistan it has been documented that more than 70% hepatitis C infections are spread in hospitals by the use of same needle several times and major or minor operations that are extremely frequent in Pakistan. Globally reuse of needles is also common source of transmission [37]. Studies show that RVR and SVR are independent of transmission routes of HCV.

### Base line diagnosis

Detection of anti HCV by ELISA is the initial step in diagnosis of HCV infection and it is more than 99% sensitive and specific [38]. PCR is the second main step in the analysis of chronic HCV infection and exposure of virus is usually detectable within 7 to 21 days [39,40]. Liver biopsy is also an important parameter in diagnosis of chronic HCV infection but as persons infected with genotype 2/3 respond well to standard therapy, treatment can be started without liver biopsy [40].

### Therapy for HCV infection

Chronic HCV is treated with a glycoprotein commonly known as interferon (INF) alpha and it is considered the backbone of therapy because it efficiently increases the immune response against virus [41]. Afterward interferon plus ribavirin become a gold standard (3 MIU thrice weekly along with ribavirin 800 to1200 mg per day). This treatment enhances SVR rate up to 38-43%. As SVR greatly depend on HCV genotype so genotype 1 needs treatment for 48 weeks to achieve SVR of 29% and genotype 2 and 3 needs treatment up to 24 weeks to attain SVR rate of 66% [42]. Currently the regular treatment of HCV is pegelated interferon (PEG-INF) in combination with ribavirin. This therapy achieves SVR of about 50% for genotype 1 and 80% for genotype 2 & 3 [43].

There are two types of pegylated interferon; PEG-IFN-alpha-2a and PEG-IFN-alpha-2b. These are dissimilar only by size and configuration of the polyethylene glycol molecules that has binding sites for interferon. The functioning of these two formulated interferon not compared still but both are equally good for HCV treatment [44].

Current HCV therapy for genotypes 2a to 2b, 3a to 3d, 5a, 6a and mixed genotypes infected patients is 3 subcutaneous injections of 3 MU of recombinant interferon alpha and ribavirin (10 mg per day per kg body weight) in one week for 6 months. Individuals infected from HCV genotype 1a to 1c, 4 and mixture of 1 and 4 HCV genotypes should receive three 3 MU subcutaneous injections of recombinant IFN alpha and ribavirin that are given orally (for individuals with ≤ 75 kg body weight) require 1,000 mg per day, for patients with > 75 kg body mass require 1,200 mg per day) in a week for total 48 weeks [45].

Conventional interferon (C-INF) therapy is used for HCV treatment in poor countries because of financial reasons and Pakistan Society of Gastroenterology and GI Endoscopy also recommend the use of C-INF therapy for HCV genotype 3 in Pakistan [46,40]. In under developed and developing countries including Pakistan, pegylated interferon therapy is beyond the reach of common poor patients [47,40]. In 2001, FDA permitted two kinds of PEG-INF (i) PEG-INF Alpha 2a (40 KD) and (ii) PEG-INF Alpha 2b (12 KD). These are administered only once a week because they have long half life of plasma (almost 10 times) in comparison with conventional INF. Liver primarily metabolizes PEG-INF Alpha 2a and kidney excretes out PEG-INF Alpha 2b. Recent studies and clinical trials confirmed that SVR rates could be increased by the using mono therapy with PEG-INF 2a or PEG-INF 2b in comparison with conventional interferon [48,40].

### Limitations of Recent HCV Therapy

It has been reported that 40% to 50% patients with HCV genotypes 1 and or 4 early attain SVR in comparison with 80% patients infected with genotypes 2 and or 3 [4,49]. However PEG-IFN and ribavirin treatment has severe side effects. Major complications of standard interferon and ribavirin therapy are anemia, cytopenias, neutropenia and thrombocytopenia as elucidated in table 1.

**Table 1:** Contraindications situations for pegylated interferon and ribavirin therapy

Contraindications levels	Situations
No more contraindications	- Regular alanine aminotransferase
	- Methadone maintenance
	- Anemia/thrombocytopenia and neutropenia
	- Restricted seizure
	- Age more than 65 years
	- Excess use of alcohol

Virtual contraindications	- Depression
	- Psychosis
	- Autoimmune disorder
	- Drug abuser
	- Renal failure (with dialysis)

Tough although not general contraindications	-Alcohol use
	-Coronary artery disorder
	- Hepatic decompensation
	- Transplantation of solid organ (except liver)

General contraindications	- Pregnancy

Novel types of interferon alpha (albinterferon) are under study; these might be very suitable anti-viral therapy because these can be given just once or twice a month as compared to standard PEG-IFN therapy [4,49]. Taribavirin, a recently introduced drug, is tested in various randomized trials that show low efficacy but also has a few complains of anemia and the side effects are easily manageable [50,4]. There are also several side affects associated with conventional interferon and ribavirin therapy including Influenza like sign and symptoms. For example headache, myalgias or arthralgias, fever, anorexia, nausea or vomiting, fatigue, abdominal pains, insomnia, suicide attempt, pruritis, anaemia, redness at injection site, dry skin, leucopoenia, irritability, thrombocytopoenia, anxiety, psychosis and laryngitis [51].

### Herbal treatment

There is no effective vaccine developed or excellent drug available for the treatment of HCV. Standard INF therapy in combination with ribavirin show sustained virological response with efficacy of not more than 50%, therefore most of the patients try herbal medicine and conventional medicine all over the world particularly in poor countries. Laccase are largely used as herbal medicine that is extracted from oyster mushroom (*Pleurotus ostreatus*). Studies showed that laccase is proficient in inhibiting the HCV replication rate [52] however the mechanism of action of this medicine is not known.

Herbal treatment can open a natural and alternative way for treatment of HCV. As Hepatitis C virus infects liver and this infection requires two or more decades to extend into substantial disease, a nutritional supplement might facilitate to decrease or stop disease development. More recent studies regarding herbal treatment provoke a hope for HCV patient that is based on a chemical known as proanthocyandin, extracted from blueberry leaves. It has been reported that proanthocyandin can stop HCV replication in infected patients [53]. According to another study rhizomes of the Chinese medicinal herb Rhodiola kirilowii may also act as possible inhibitor of HCV [54].

### Factors affecting treatment response

Treatment response is better in patient of less than 40 years of age in comparison with elderly. Young females respond well to the treatment. High intensity of viremia is related with deprived response. Immunodeficiency, excessive use of alcohol and co-infection with HIV or HBV, all harmfully cause the result to HCV infection [55,16].

HCV therapy is not suitable for people suffering from severe HCV related cirrhosis, undergone organ transplant, children of < 3 years and specific contraindication to the medication. Interferon causes severe side effect includes, anxiety, irritability personality changes, even suicide, depression or acute psychosis. Ribavirin side effect included anemia, renal dysfunction of coronary artery. Fetal abnormality and fatality are important side effects of ribavirin, a well-known teratogen.

Due to the distinctive character of the virus to develop vaccine against HCV leftovers, a disappointment has been seen due to its high mutation rate. It has already been reported that the rate of HCV reproduction is high and the error-prone polymerase causes mutation continuously. The high HCV replication rate provides sufficient chance of mutation that occurs in the viral population inside an infected person. Production of virus has been estimated at 10^12 ^(one trillion) new HCV virions per day [56]. Studies on chronically infected HCV patients show that rate of mutation in HCV genome has been approximately 0.001 substitutions per genomic site in one year. Such high rate of mutation could result into 8-18 mutations within the RNA of 9.6 kb genomic size. It has also been reported that envelop protein E2 has highly mutated sites known as hypervariable region HVR1. High variation in E2 causes immune escape mutants of the virus as of the neutralizing antibodies and therefore describes the constant viremia. In addition to E2 gene, P7 region has also been shown with increased variability [16].

### Future perspectives

New therapeutic approaches are under study like interferon related systems, modified forms of ribavirin, siRNA, internal ribosome entry site (IRES) inhibitors, NS3 and NS5a inhibitors and novel immunomodulators. These are particularly for those patients who show low SVR rate by traditional therapies. More remedial therapies include antifibrotic agents, caspase inhibitors and antibody treatment and vaccines. Particularly targeted antiviral compounds like specifically targeted anti-viral therapy for hepatitis C' (STAT-C) compounds are now under study by scientists that are used along with standard interferon therapy. Reports confirm improved SVR rate at least in HCV genotype 1 patients. Further studies are required to confirm its significance in the clearance of HCV RNA if used as a single therapy without interferon and ribavirin [57,58].

## Conclusion

Currently chronic HCV treatment consists of pegelated interferon alpha and a nucleoside analogue ribavirin for 3 to 18 months. However several side effects are associated with this treatment. New therapeutic approaches are under study and recent clinical trials are being focused on inhibitors of HCV NS3 and NS5a RNA polymerase. Parameters that increase SVR rate for HCV are genotype 2 and 3, age < 40 years and low viral load before treatment.

## Abbreviations

HCV: hepatitis C virus; PEG-INF: pegylated interferon; RVR: rapid virological response; SVR: sustained virological response; RBV: ribavirin; ETR: end of treatment response; ELISA: enzyme linked immunosorbant assay; PCR: polymerase chain reaction; MIU: million international units; SDINF: standard interferon; HVR: hiper variable region; IRES: internal ribosome entry site; STAT-C: specifically targeted anti-viral therapy for hepatitis C.

## Competing interests

The authors declare that they have no competing interests.

## Authors' contributions

SM and SS reviewed the literature, and wrote the manuscript. MI edited the manuscript. AT, SB, BR, AH, SB, ZA, MN, ZF, MA, LA, MA, MA, BK, helped SM & SS in literature review. All the authors read and approved the final manuscript.
